# A Clinical Study on Glucosamine Sulfate versus Combination of Glucosamine Sulfate and NSAIDs in Mild to Moderate Knee Osteoarthritis

**DOI:** 10.1100/2012/902676

**Published:** 2012-04-01

**Authors:** Tamil Selvan, Kingston Rajiah, M. Sundara-Moorthi Nainar, Elizabeth M. Mathew

**Affiliations:** ^1^Department of Pharmacy Practice, Chalapathi Institute of Pharmaceutical Sicences, Guntur-522034, India; ^2^Department of Pharmacy Practice, International Medical University, no. 126, Jalan Jalil Perkasa 19, Bukit Jalil, Kuala Lumpur-57000, Malaysia; ^3^Department of Pharmacokinetics, Lupin Bioresearch Center, Pune-411021, India; ^4^Department of Pharmacy Practice, Mallige College of Pharmacy, Bangalore-560090, India

## Abstract

*Background*. Glucosamine may be effective in treating and possibly slowing the progression of Osteoarthritis (OA). It is believed Glucosamine supplements may help to stop cartilage breakdown, build cartilage and decrease swelling. *Objective*. The objective of this study was glucosamine sulfate versus combination of glucosamine sulfate and Non-Steroidal anti-inflammatory drugs (NSAID) in mild to moderate knee osteoarthritis. *Methods*. Subjects were randomly recruited from Rheumatology outpatient department after a diagnosis of mild or moderate Osteoarthritis. Study tools like patient data collection form, Western Ontario McMaster Universities Arthritis index (WOMAC) of Osteoarthritis questionnaires and Visual Analog Scale (VAS) were used. *Results*. After 12 weeks, WOMAC total score the result showed that the significant mean difference between the group A and Group B treatment (*P* < 0.01), with a combination of GS and NSAIDs reducing VAS pain scores. Thus, it is found that Group B treatments over 4 and 12 weeks produced improved WOMAC and VAS grades. *Conclusions*. Study results may suggest that the Glucosamine Sulfate has a carryover effect like Disease modifying agents. Long-term treatment of Glucosamine Sulfate may reduce the dependence of NSAIDs usage and delay the disease progression. Thereby we can reduce the NSAIDs side effects and improve the patient's quality of life.

## 1. Introduction

Pharmacological treatment of osteoarthritis can be divided into two groups: symptom-modifying and disease-modifying drugs [1]. Symptom-modifying drugs are at present the prescription of choice for patient with osteoarthritis, for example, NSAIDs. However, they are also the case of serious side effects [2, 3]. Disease-modifying agents are not yet available in usual care. Of most biological agents, glucosamine sulfate seems to be most promising [4, 5]. Glucosamine, which occurs naturally in the body, plays a key role in the construction of cartilage (the tough connective tissue that cushions the joints). Glucosamine is the most fundamental building block required for biosynthesis of the classes of compounds including glycolipids, glycoproteins, hyaluronate, and proteoglycans [6, 7]. As a component of these macromolecules, glucosamine has a role in the synthesis of cell membrane, lining, collagen, osteoid, and bone matrix. Glucosamine is also required for the formation of lubricants and protective agents such as mucin and mucous secretion [8, 9].

**Objectives**

The objectives of this study are to determine the effectiveness of Glucosamine sulfate in reducing joint pain in mild to moderate knee OA, to assess the effectiveness of glucosamine sulfate in improving joint physical function in mild to moderate knee OA, and to ensure the therapeutic efficacy and safety of glucosamine sulfate as a disease-modifying agent in osteoarthritis compared with a combination of glucosamine sulfate and nonsteroidal anti-inflammatory drug in mild to moderate knee OA.

## 2. Materials and Methods

### 2.1. Study Population

This study includes male and female patients in the age group more than 20 years. The subjects who were able to provide written informed consent have been included in the study. Rheumatoid arthritis patients and the patients with joint replacement in knees were excluded. Also, pregnant and lactating women as well as the patients having a history of chronic infection such as hepatitis and COPD were excluded [10]. Study subjects were randomly recruited from Rheumatology Outpatient Department after a diagnosis of mild or moderate osteoarthritis. In total, 143 patients were interviewed, 100 qualified, and 82 completed the study. Group A consists of 43 patients and group B consists of 39 patients. Group A patients were treated with glucosamine sulfate (GS) 500 mg t.i.d and reviewed on every 30 days once for 3 months. Group B patients were treated with glucosamine sulfate 500 mg t.i.d along with any one of the conventional NSAIDs (Ibuprofen or Piroxicam) and reviewed on every 30 days once for 3 months.

### 2.2. Statistical Methods

Documented datas were entered into SPSS.PC version 8 [11]. Within the group, variables were compared with paired *t*-test. Between the groups, variables were compared with independent *t*-test. Statistical significance was taken at the 95% level (*P* < 0.05). Results were expressed as mean ± standard deviation [12].

### 2.3. Source of Data

Baseline demographic information like sex, age, comorbidities, duration of osteoarthritis and medication history were collected from patient's case sheet, patient's medication chart, and direct interview [13]. Study tools like patient data collection form, Western Ontario McMaster Universities Arthritis index (WOMAC) Osteoarthritis questionnaires [14], and visual analog scale (VAS) were used [15].

### 2.4. Outcome Measures

#### 2.4.1. Primary Efficacy Variables

The western Ontario and McMaster universities (WOMAC) osteoarthritis index is a disease-specific self-administered health status measure that is widely accepted as reflective of Osteoarthritis disease activity [16]. The original index consists of 24 Questions (5 pain, 2 stiffness and 17 physical function). Individual question response is assigned a score of between 0 (none) to 4 (extreme) and summed to form a score ranging from 0 (best) to 96 (worst). There are three sections to the WOMAC score; section A deals with the amount of pain (5 questions), section B address the amount of joint stiffness (2 questions), section C address aspects of physical function (17 questions).

#### 2.4.2. Secondary Efficacy Variables

VAS-visual analog scale score uses a 100 mm linear measure of pain status with 0 representing no pain and 100 being unbearable pain [17]. Patients marked on the linear scale the relevant amount of pain they were experiencing, and the value was noted.

## 3. Results and Discussion

In total, 143 patients were interviewed. 100 subjects with OA of the knee were randomized and divided into two groups. All subjects received trial medication immediately after randomization [18]. 18 subjects were dropped out form this study because they were lost to followup and refused further therapy [19], 8 subjects due to poor compliance (3 GS, 5 GS + NSAID), 6 due to gastrointestinal upset (GS + NSAID), and 4 due to inadequate pain control (GS) [20]. Finally, 82 subjects completed the study: group A (GS, *n* = 43), group B (GS + NSAID, *n* = 39). Among the study subjects, the mean age of the female subjects (47.96 ± 5.09 and 48.95 ± 8.94) was lower than the male subjects in groups A and B. This result indicates females are affected by knee OA much earlier than male [21]. Out of 82 subjects studied, the percentage of female subjects was greater than the percentage of male subjects, that is, 60.46% and 53.84%, respectively in group A and group B. This result showed that females are more prevalent to knee OA [22]. The subjects with the age groups of 41–50 years and 51–60 years were the highest in number by 21 (48.83%) and 19 (44.18%) in group A and 14 (35.89%) in group B. 2 (4.65%) subjects in the age group of less than 40 years were noted in group A but 0% in group B were reported during the study period. Among these patients, 10 (23.25%), 5 (12.82%) were affected by left knee OA, 11 (25.58%), 12 (30.76%) were affected by right knee OA, and 22 (51.62%), 22 (56.41%) were affected by bilateral knee OA in groups A and B. This result clearly indicates the most of the subjects affected by bilateral knee OA [23].

 The mean body mass index (BMI) ratio of subjects was noted under the overweight category [24]. In group A, 25.6 was noted in both male and female. In group B, 25.83 and 26.48 were noted in males and females, respectively. This result showed that the overweight people are more promptly affected by knee OA [25].

### 3.1. Primary Efficacy Variables Data

This data revealed that the mean WOMAC pain score of group A was 16.83 ± 1.68 on 0 week (*n* = 43) and 17.5 ± 0.93 after 4 weeks. The mean difference was not statistically significant. But, after 4 weeks, the mean WOMAC pain score was 10.58 ± 0.58 with the mean difference of 6.25 ± 1.83. This mean score decrease was statistically highly significant (*P* < 0.01). The mean WOMAC pain score of group B was 18.17 ± 1.84 on 0 week (*n* = 39) and 13.3 ± 2.56 after 4 weeks.

The difference was statistically significant (*P* < 0.01). After 12 weeks, the mean WOMAC pain score was 5.20 ± 1.12 with the mean difference of 12.97 ± 2.15 (Table 2). This result revealed that the score decrease was statistically highly significant (*P* < 0.01) [26].

 Between the groups analysis, the results showed the mean difference was 4.20 (95% confident interval (CI) 3.33 to 5.03) on first review (*P* < 0.01). The men difference was 5.37 (95% confident interval 4.97 to 5.78) on last review (after 12 weeks) see Table 1. These results revealed the significant mean difference between group A and group B (*P* < 0.01) [27].

The mean WOMAC stiffness score of group A was 6.37 ± 0.69 on 0 week (*n* = 43) and 6.25 ± 0.49 after 4 weeks. The decrease was not statistically significant.

But, after 12 weeks, the mean WOMAC stiffness score was 4.00 ± 0.30 with the mean difference of 2.37 ± 0.69. This result revealed that the mean score decrease was statistically significant (*P* < 0.01). The mean WOMAC stiffness score of group B was 6.64 ± 0.95 on 0 week (*n* = 39) and 4.89 ± 0.64 after 4 weeks. The decrease was statistically significant (*P* < 0.01). But, after 12 weeks, the mean WOMAC stiffness score was 1.76 ± 0.58 with the mean difference of 4.87 ± 0.62. This result revealed that the score decrease was statistically highly significant (*P* < 0.01).

Between the groups analysis, results showed the mean difference was 1.35 (95% confident interval 1.10 to 1.61) on first review (*P* < 0.01). The mean difference was 2.23 (95% confident interval 2.02 to 2.44) after 12 weeks, see Table 2. These results revealed the significant mean difference between group A and group B (*P* < 0.01) [28].

The mean WOMAC function score of group A was 24.51 ± 2.17 on 0 week (*n* = 43) and 29.25 ± 3.43 after 4 weeks. The mean decrease was not statistically significant. But, after 12 weeks, the mean WOMAC function score was 16.02 ± 1.14 with the mean difference of 8.48 ± 2.93. This result revealed that the mean score decrease was statistically highly significant (*P* < 0.01). The mean WOMAC function score of group B was 25.58 ± 2.37 on 0 week (*n* = 39) and 21.69 ± 2.36 after 4 weeks. The mean decrease was statistically significant (*P* < 0.01). After 12 weeks, the mean WOMAC function score was 7.82 ± 1.86 with the mean difference of 17.76 ± 2.59. This result revealed that the mean score decrease was statistically highly significant (*P* < 0.01).

 Between the groups analysis, results showed the mean difference was 7.56 (95% confident interval −1.83 to 16.95) on first review (*P* < 0.01). The men difference was 8.20 (95% confident interval 7.51 to 8.87) after 12 weeks, see Table 3. These results revealed the significant difference between the group A and group B (*P* < 0.01) [29].

The mean WOMAC total score of group A was 47.65 ± 3.69 on 0 week (*n* = 43) and 48.09 ± 2.23 after 4weeks. No change was observed by 4 weeks. But, after 12 weeks, the mean WOMAC total score was 30.58 ± 1.41 with the mean difference of 17.06 ± 4.54. This result revealed that the mean score decrease was statistically highly significant (*P* < 0.01). The mean WOMAC total score of group B was 50.76 ± 3.88 on 0 week (*n* = 39) and 39.64 ± 4.31 after 4 weeks. The decrease was statistically significant (*P* < 0.01). But, after 12 weeks, the mean WOMAC total score was 14.79 ± 3.16 with the mean difference of 35.97 ± 4.24. This result revealed that the mean score decrease was statistically highly significant (*P* < 0.01).

 Between the groups analysis, results showed the mean difference was 8.45 (95% confident interval 6.90 to 9.99) on first review (*P* < 0.01). The mean difference was 15.78 (95% confident interval 14.68 to 16.89) on last review (after 12 weeks), see Table 4. These results revealed the significant difference between group A and group B (*P* < 0.01) [30].

### 3.2. Secondary Efficacy Variables Data

Analysis of Group A (*n* = 43) showed that the mean VAS pain score was 8.65 ± 0.74 on 0 week and 8.7 ± 0.40 on after 4 weeks. The mean difference was not statistically significant in first review. But after 12 weeks the mean VAS pain score was 6.39 ± 0.72 with the mean difference of 2.25 ± 0.84. This result clearly indicates the mean decrease in pain score was statistically significant (*P* < 0.01).

Analysis of Group B (*n* = 39) showed that the mean VAS pain score was 9.21 ± 0.62 on 0 week and 7.91 ± 0.62 on after 4 weeks. The difference was not statistically significant in first review. But, after 12 weeks, the mean VAS pain score was 5.26 ± 0.57 with the mean difference of 3.94 ± 0.62. This result clearly indicates the mean decrease in pain score was statistically significant (*P* < 0.01).

 Between the groups analysis, results showed the mean difference was 0.79 cm (95% confident interval 0.56 to 1.01) on first review (*P* < 0.01). The mean difference was 1.12 (95% confidence interval 0.84 to 1.41) after 12 weeks, see Table 5.

This result revealed the significant mean difference between group A and group B treatment (*P* < 0.01), with a combination of GS and NSAIDs reducing VAS pain scores.

## 4. Conclusions 

This study demonstrates the efficacy of glucosamine sulfate (GS) compared with a combination of glucosamine sulfate and nonsteroidal anti-Inflammatory drugs (NSAIDs) in Mild to Moderate Knee osteoarthritis Patients based on the comparison of WOMAC Pain, stiffness, physical function, total score, and VAS pain score. The results from the first review revealed that the combination of GS with NSAIDs showed better improvement in pain, stiffness and physical function compared with glucosamine sulfate alone group. After the final review, results revealed that the GS group also has significant improvement in pain, stiffness and physical function but lesser compared with GS and NSAIDs group. This study results may suggest that the Glucosamine Sulfate has a carryover effect like disease modifying agent. However, long-term treatment of glucosamine sulfate may reduce the dependence of NSAID usage and delay the disease progression. Thereby, we can reduce the NSAIDs side effects and improve the patient's quality of life.

## Figures and Tables

**Table 1:** Comparison of WOMAC pain mean score between group A and group B.

Review	Mean difference	95% confidence interval of the difference		*t*-value
		Lower	Upper	
0 week	1.3423	2.1234	0.5611	3.421*
4 weeks	4.2039	3.3288	5.079	9.664*
8 weeks	4.3703	3.7113	5.0293	13.204*
12 weeks	5.3763	4.9724	5.7801	26.669*

*P < 0.01.

**Table 2:** Comparison of WOMAC stiffness mean score between group A and group B.

Review	Mean difference	95% confidence interval of the difference		*t*-value
		Lower	Upper	
0 week	0.2689	0.6406	0.0960	1.444^NS^
4 weeks	1.3584	1.1049	1.1611	10.686*
8 weeks	1.6971	1.1414	1.9792	12.015*
12 weeks	2.2308	2.2130	2.4402	21.333*

*P < 0.01, NS: Not significant.

**Table 3:** Comparison of WOMAC function mean score between group A and group B.

Review	Mean difference	95% confidence interval of the difference		*t*-value
		Lower	Upper	
0 week	1.0781	2.0817	0.0745	2.139^NS^
4 weeks	7.5635	1.8305	16.9575	1.624^NS^
8 weeks	5.8968	5.0456	6.7481	13.797*
12 weeks	8.2027	7.5122	8.8933	23.745*

*P < 0.01, NS: Not significant.

**Table 4:** Comparisons of WOMAC total mean score between group A and group B.

Review	Mean difference	95% confidence interval of the difference		*t*-value
		Lower	Upper	
0 week	3.1181	4.7903	1.4458	3.712*
4 weeks	8.452	6.9091	9.9949	10.975*
8 weeks	11.7847	10.4513	13.1181	17.603*
12 weeks	15.7865	14.681	16.8921	18.659*

*P < 0.01.

**Table 5:** Comparisons of VAS Pain mean score between group A and group B.

Review	Mean difference	95% Confidence Interval of the difference		*t*-value
		Lower	Upper	
0 week	−0.5668	0.8682	0.2654	3.743*
4 weeks	0.7897	0.5625	1.017	6.936*
8 weeks	0.8927	0.5841	1.02013	5.764*
12 weeks	1.1261	0.8369	1.4127	7.824*

*P < 0.01.
